# AMPK–a key factor in crosstalk between tumor cell energy metabolism and immune microenvironment?

**DOI:** 10.1038/s41420-024-02011-5

**Published:** 2024-05-18

**Authors:** Na Wang, Bofang Wang, Ewetse Paul Maswikiti, Yang Yu, Kewei Song, Chenhui Ma, Xiaowen Han, Huanhuan Ma, Xiaobo Deng, Rong Yu, Hao Chen

**Affiliations:** 1 The Second Clinical Medical School, Lanzhou University, Lanzhou, Gansu 730030 China; 2 The Department of Tumor Surgery, The Second Hospital of Lanzhou University, Lanzhou, Gansu 730030 China; 3 Key Laboratory of Environmental Oncology of Gansu Province, Lanzhou, Gansu 730030 China

**Keywords:** Cancer, Diseases

## Abstract

Immunotherapy has now garnered significant attention as an essential component in cancer therapy during this new era. However, due to immune tolerance, immunosuppressive environment, tumor heterogeneity, immune escape, and other factors, the efficacy of tumor immunotherapy has been limited with its application to very small population size. Energy metabolism not only affects tumor progression but also plays a crucial role in immune escape. Tumor cells are more metabolically active and need more energy and nutrients to maintain their growth, which causes the surrounding immune cells to lack glucose, oxygen, and other nutrients, with the result of decreased immune cell activity and increased immunosuppressive cells. On the other hand, immune cells need to utilize multiple metabolic pathways, for instance, cellular respiration, and oxidative phosphorylation pathways to maintain their activity and normal function. Studies have shown that there is a significant difference in the energy expenditure of immune cells in the resting and activated states. Notably, competitive uptake of glucose is the main cause of impaired T cell function. Conversely, glutamine competition often affects the activation of most immune cells and the transformation of CD4^+^T cells into inflammatory subtypes. Excessive metabolite lactate often impairs the function of NK cells. Furthermore, the metabolite PGE2 also often inhibits the immune response by inhibiting Th1 differentiation, B cell function, and T cell activation. Additionally, the transformation of tumor-suppressive M1 macrophages into cancer-promoting M2 macrophages is influenced by energy metabolism. Therefore, energy metabolism is a vital factor and component involved in the reconstruction of the tumor immune microenvironment. Noteworthy and vital is that not only does the metabolic program of tumor cells affect the antigen presentation and recognition of immune cells, but also the metabolic program of immune cells affects their own functions, ultimately leading to changes in tumor immune function. Metabolic intervention can not only improve the response of immune cells to tumors, but also increase the immunogenicity of tumors, thereby expanding the population who benefit from immunotherapy. Consequently, identifying metabolic crosstalk molecules that link tumor energy metabolism and immune microenvironment would be a promising anti-tumor immune strategy. AMPK (AMP-activated protein kinase) is a ubiquitous serine/threonine kinase in eukaryotes, serving as the central regulator of metabolic pathways. The sequential activation of AMPK and its associated signaling cascades profoundly impacts the dynamic alterations in tumor cell bioenergetics. By modulating energy metabolism and inflammatory responses, AMPK exerts significant influence on tumor cell development, while also playing a pivotal role in tumor immunotherapy by regulating immune cell activity and function. Furthermore, AMPK-mediated inflammatory response facilitates the recruitment of immune cells to the tumor microenvironment (TIME), thereby impeding tumorigenesis, progression, and metastasis. AMPK, as the link between cell energy homeostasis, tumor bioenergetics, and anti-tumor immunity, will have a significant impact on the treatment and management of oncology patients. That being summarized, the main objective of this review is to pinpoint the efficacy of tumor immunotherapy by regulating the energy metabolism of the tumor immune microenvironment and to provide guidance for the development of new immunotherapy strategies.

## Facts

1. The dynamic network of cell metabolism undergoes changes throughout tumor progression, exerting profound effects on the activity, state, and function of both tumor cells and immune cells.
2. As a sensitive energy receptor and regulator in eukaryotes, AMPK is intricately associated with various bioenergetic processes and the anti-tumor immune response during tumor development. It serves as a metabolic mediator that connects tumor energy metabolism with the immune microenvironment, thereby providing novel insights into cancer immunotherapy.
3. AMPK acts as a “double-edged sword” by not only regulating the metabolic state of tumor cells but also enhancing their internal metabolism’s diversity and flexibility. This ultimately leads to increased drug resistance and treatment challenges. Moreover, AMPK plays a crucial role in modulating the metabolic state of immune cells, enabling them to sustain their activity and function effectively. Consequently, it reduces tumor metastasis while enhancing the efficacy of immunotherapy. Therefore, investigating optimal timing for activating AMPK and identifying mechanisms that can maximize its beneficial effects on tumor therapy hold promise as an immunotherapy strategy.

## Open questions

1. In which stage of tumor progression is AMPK most optimally upregulated to activate the immune system for efficient eradication of tumor cells?
2. What are the specific mechanisms through which AMPK activates anti-tumor immunity via energy metabolism pathways?
3. Apart from the core factor AMPK in energy metabolism, what other factors can synergistically contribute to metabolic reprogramming of the tumor immune microenvironment and exert anti-tumor effects?

## Introduction

Cancer poses a significant threat and burden to human health in present-day society, emerging as a major global public health concern. It is worth mentioning that cancer stands as the second leading cause of death, with a staggering 19,292,789 new cases and 9,958,133 new deaths reported worldwide in 2020 [1].

Currently, tumor immunotherapy has emerged as a crucial research area in therapy strategy, which attacks and eliminates tumor cells by boosting or stimulating the patient’s own immune system [2]. Moreover, tumor immunotherapy has yielded remarkable results in multiple clinical trials, effectively controlling various types of tumors, including melanoma [3], non-small cell lung cancer [4], breast cancer [5], and gastric cancer [6]. The prevailing methods utilized in tumor immunotherapy encompass immune checkpoint inhibitors (ICIs), CAR T cell therapy, cytokine therapy, tumor vaccines, T cell enhancers, and others.

In particular, ICIs therapy based on monoclonal antibodies has been widely used as the first-line treatment of a variety of solid tumors. To date, all approved ICIs are monoclonal antibodies that block cytotoxic T lymphocyte-associated protein 4 (CTLA-4), programmed cell death protein-1 (PD-1), or programmed cell death ligand 1 (PD-L1), all of which are key inhibitors of T cell activation and function. Since ipilimumab was approved by FDA for metastatic melanoma in 2011 [7], various ICIs have been developed successively, and multiple studies have shown that ICI has shown benefits in non-small cell lung cancer [8], liver cancer [9], triple-negative breast cancer [10], gastroesophageal cancer [11], ovarian cancer [12] and other malignant tumors. Nevertheless, some patients do not respond to ICIs therapy, and many patients who initially respond to therapy eventually relapse due to acquired resistance. The current objective response rate for ICIs is 30% or less [13]. Some of these patients experienced false progression (overall incidence 2-11% [14]) during ICIs treatment, while others experienced hyperprogression. False progression and hyperprogression are unique phenomena of ICIs treatment. If false progression is missed, it interferes with patients’ normal treatment cycle, and patients are more likely to suffer immune-related adverse events. In the absence of judgment of hyperprogression, the use of ICIs accelerates the progression of the tumor, leading to a poor prognosis for the patient. Why is ICIs treatment more effective in limited cancer patients and less effective or even ineffective in most cancer patients?

The reasons for the limited effectiveness of tumor immunotherapy can be attributed to the following factors; 1) Tumor heterogeneity: Different tumor cells may possess various surface markers that can be recognized and attacked by the immune system. However, these markers can differ among patients, presenting challenges for the immune system to effectively identify and target tumor cells [15]. 2) Immune tolerance: Tumor cells often employ a variety of mechanisms to suppress the attack of the immune system, striving to achieve the effect of immune escape. For example, tumor cells can reduce the expression of tumor antigens, release signaling molecules that hinder immune cells, or change the environment around tumor cells (promoting remodeling of the vasculature and extracellular stroma) to evade immune attack [16, 17]. 3) Peripheral immune escape: Tumor cells can secrete some immunosuppressive substances (IL-10, IL-4, TGF-β), thereby affecting the activity and immune response of peripheral immune cells, thus limiting the effect of immunotherapy [17]. 4) Immunosuppressive environment: there are some immunosuppressive cells and factors in tumor tissues, including regulatory T cells (Treg) and inhibitory secretory factors, such as IL-4, IL-10, and TGF-β. Tumor cells also hijack immune cells (such as neutrophils, macrophages, and Treg cells) to orchestrate the immunosuppressive TIME (Tumor immune microenvironment) [18]. 5) Other factors: Immunotherapy is also affected by many other factors, such as the patient’s immune status, tumor course, treatment approaches, and so on. Therefore, in order to enhance the success rate of tumor immunotherapy, it is crucial to better understand the mechanism of tumor immune escape, identify more targets for immunotherapy, and combine immunotherapy with other therapeutic methods.

The problem arises because the tumor environment is a complex ecosystem in which interactions between tumor cells and host cells influence disease progression and therapeutic response. TIME (Tumor immune microenvironment) consists of not only tumor cells, but also various types of immune cells, cancer-associated fibroblasts, endothelial cells, pericytes, and other tissue-resident cells [2]. These host cells were once thought to be bystanders to tumorigenesis, but they are now known to play a key role in the pathogenesis of tumors. Apart from tumor cells, immune cells are arguably and undeniably the most complex actors in solid tumors, and their activity can range from antitumorigenic to tumorigenic [18]. The cell composition and functional state of TIME can be greatly different due to the organ affected by the tumor, the intrinsic characteristics of the tumor cells, the tumor stage, and the characteristics of the patient. The energy metabolism level of TIME can directly or indirectly affect the activity and function of immune cells, thus affecting the progress of the tumor and the efficacy of immunotherapy. Although angiogenesis in TIME is increased, it fails to meet the demand for nutrients and oxygen of infinite proliferating tumor cells, resulting in an acidic microenvironment lacking sugar and oxygen, making the surrounding immune cells short of glucose, oxygen, and nutrients [19, 20]. Such an environment has resulted in a decrease in the activity of immune cells and an increase in immunosuppressive cells. Tumor cells typically exhibit a metabolic characteristic known as aerobic glycolysis (Warburg effect), which speeds up energy supply but results in a low energy utilization rate [21]. Moreover, many immune cells require multiple metabolic pathways to maintain their normal function and activity. For example, cellular respiration and oxidative phosphorylation pathways can be used to produce reactive oxygen and essential metabolites required for immune recognition [22, 23]. In addition to the influence of the lesion area, genetic factors, other metabolic diseases such as obesity and diabetes, and systemic conditions such as diet structure will also affect the metabolic reprogramming of the tumor development process. Furthermore, the tumor’s own organizational structure, resident cell types, epigenetic patterns, and transcriptional networks, as well as the internal phenotype of cancer cells, will also affect the metabolic reprogramming of tumor development. Factors like cancer genotype, interference signal, and gene expression cannot be ignored. During immunotherapy, in order to improve the activity of immune cells, and enhance immune monitoring and attack ability, it may be necessary to alter the metabolic characteristics of tumor cells as well as immune cells. Metabolic reprogramming affects the occurrence and development of tumors, and the metabolic changes of tumor cells can influence TIME, which in turn affects the immune response, which also reveals that TIME can be reshaped through targeted metabolic pathways, thereby benefiting immunotherapy.

Metabolic reprogramming may regulate TIME through the following aspects: (1) Cellular glycolysis: Tumor cells tend to produce ATP through aerobic glycolysis. At the same time, aerobic glycolysis can also increase a large amount of metabolic waste, such as lactic acid, ketone bodies, and pyruvate, which can affect the activity and migration of immune cells [24]. (2) Cytochrome oxidase system: Cytochrome oxidase contains five types of enzymes that can promote intracellular REDOX reaction. These enzymes can utilize intracellular formic acid as a substrate to catalyze REDOX reaction, transfer electron energy and produce antioxidant substances such as thioacetylcystine. In TIME, the activity of cytochrome oxidase will increase to respond to the excessive REDOX state, counteract excessive reactive oxygen species, and enhance its antioxidant capacity [25]. (3) Cell respiratory chain system: Mitochondria are the main energy supply center in the cell, they are involved in productivity and cell respiration and serve as an energy source for immune cells to carry out phagocytosis. In TIME, if the mitochondrial function is abnormal, it will not only inhibit the growth of tumor cells but also restrain the growth and function of immune cells, thereby affecting the normal function of the immune system in the body [26]. Therefore, to correct the abnormalities of cell glycolysis, cytochrome oxidase, and cell respiratory chain system by TIME, explore strategies to intervene in tumor metabolism, which is expected to help improve the function of immune cells in TIME, thus improving the effect of tumor immunotherapy. Metabolic reprogramming is not only very important in the progression of tumors, but also in precancerous lesions, many specific metabolic reprogramming will occur in the lesion area, offering great possibilities for the development of biomarkers for screening early lesions. Previous studies have targeted atypical adenomatoid hyperplasia (AAH) of the lung. Overexpression of sodium-dependent glucose transporter 2 (SGLT2) in premalignant lesions has been developed as a cancer imaging tracer [27]. Research on epigenetic enzymes in leukemia has found that metabolic markers of early metabolic reprogramming in cancer lesions have targeted therapeutic functions and significance [28].

AMPK (AMP-activated protein kinase) functions as an enduring energy sensor and regulator within eukaryotic cells, existing as a heterotrimeric complex composed of three distinct αβγ subunits encoded by separate genes [29]. Twelve different isoforms have been identified thus far, each exhibiting unique subcellular localization and functionality with intricate associations with tumorigenesis and therapeutic interventions [30]. Unraveling strategies that optimize AMPK’s anticancer effects while inhibiting its protumorigenic actions remains a focal point for research endeavors. AMPK can be activated through various mechanisms including LKB1- or CaMKKb-mediated phosphorylation events, direct sensing of ATP/ADP ratio fluctuations, pharmacological agents such as metformin and AICAR administration, small molecule compounds like A-769662 application, exercise-induced stimuli or hypoxic conditions [31, 32]. As a key orchestrator of metabolic regulation encompassing diverse aspects such as carbohydrate lipoprotein metabolism along with mitochondrial dynamics control over ribosomes and nucleic acids homeostasis alongside autophagy modulation plus cell cycle regulation governing proliferation/apoptosis decisions - AMPK exhibits multifaceted roles in cellular processes. AMPK not only directly inhibits biosynthetic pathways such as lipids, glycogen, and rRNA to inhibit cell growth but also indirectly inactivates the mechanistic target of rapamycin complex-1, a key signaling node that promotes cell growth [33]. Additionally, AMPK induces G1 cell cycle arrest to further suppress cell proliferation. Given these potential tumor-suppressive effects, it is widely believed that AMPK exerts its function as a tumor suppressor through its upstream kinase LKB1 [34]. Supporting this idea, studies suggest that whole-body knockout of AMPKα1 in mice accelerates lymphoma development driven by Myc oncogene overexpression in B cells [35]. Furthermore, recent findings from various in vitro and in vivo models demonstrate that knocking down or knocking out AMPK reduces tumor cell growth or viability [36]. The functional differences of AMPK observed across different cancer models highlight its importance as a target for personalized precision medicine development.

**Fig. 1:**
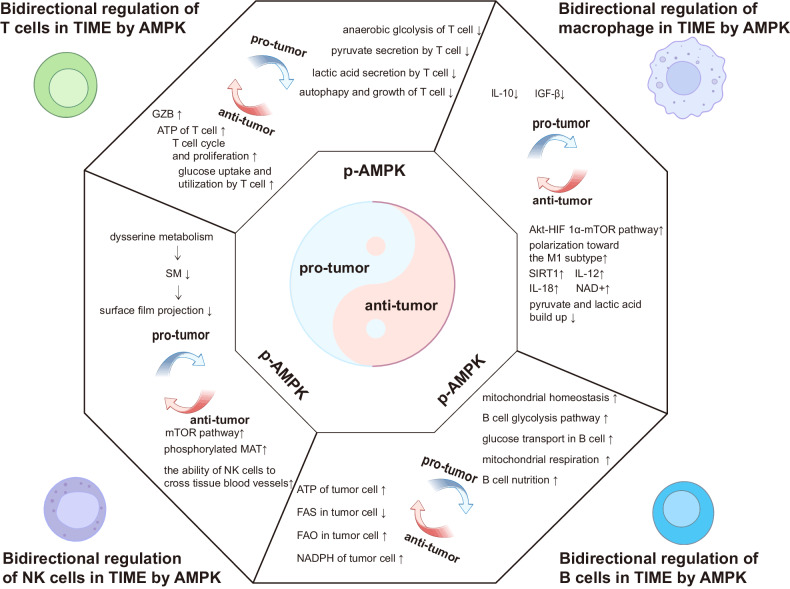
Bidirectional regulation mechanism of AMPK at TIME. AMPK exerts bidirectional regulation in the tumor immune microenvironment, impacting not only tumor cells but also immune cells. For instance, T cells are subjected to glucose uptake and utilization by T cell, ATP of T cell, GZB and T cell cycle and proliferation to suppress tumors and anaerobic glcolysis of T cell, pyruvate secretion by the T cell, lactic acid secretion by T cell, autophapy and growth of T cell to promote tumorigenesis. Macrophages undergo Akt-HIF 1α-mTOR pathway, polarization toward the M1 subtype, pyruvate and lactic acid build up for tumor inhibition, and IL-10, IGF-β for tumor promotion. B cells exhibit tumor suppression with mitochondrial homeostasis of B cell, B cell glycolysis pathway, glucose transport in B cell, mitochondrial respiration of B cell and promotion with ATP of tumor cell, FAS in tumor cell, FAO in tumor cell, NADPH of tumor cell. NK cells inhibit tumors through mTOR pathway, phosphorylated MAT, the ability of NK cells to cross tissue blood vessels while promoting tumorigenesis via SM and surface film projection.

As a core factor of stably expressed energy metabolic processes in eukaryotes, activation of AMPK is critical for alleviating metabolic and energy stress associated with tumor progression. More and more studies have demonstrated that AMPK can regulate energy metabolism through multiple pathways and mechanisms, and ultimately impact TIME, such as: 1. Metabolic adaptability: AMPK can regulate the energy metabolism of tumor cells by promoting glycolysis and fatty acid oxidation (FAO), which affects the ATP synthesis capabilities of tumor and immune cells. Such changes in energy metabolism will affect the composition of TIME and the differentiation and function of different cells [29]. AMPK directly phosphorylates PFKFB2 and PFKFB3 isoforms of fructose-2-kinase/fructose-2, 6-bisphosphatase [37] to stimulate glycolytic flux. It also inhibits cholesterol synthesis by phosphorylating the rate-limiting enzyme 3-hydroxy-3-methylglutaryl-Coenzyme A (HMG-CoA) reductase (HMGCR). Activation of AMPK leads to inhibition of HMGCR involved in cholesterol synthesis and hormone-sensitive lipase (HSL) responsible for preventing lipolysis [38]. Moreover, AMPK plays an important role in accelerating lipid catabolism by promoting fatty acid transport to mitochondria where they undergo beta oxidation mediated by carnitine palmitoyltransferase-1 (CPT-1) [39]. 2. Immune cell activity: AMPK can regulate the activity of immune cells such as macrophages, T cells, and NK cells to enhance their killing ability on tumor cells [32]. Such as, AMPK deacetylates SIRT1 and other proteins through NAD^+^ to inhibit HIF-1a and NF-kB activities while influencing M1/M2 polar mitochondrial biogenesis in macrophages [40]. Rao et al. demonstrated that AMPK regulates protein phosphatase activity, thereby governing the survival and function of CD8^+^ T cells, thus augmenting their role in tumor immune surveillance [41]. Braverman et al. reported that increased AMPK activity coordinates oxidative metabolism, proliferation, and in vitro recovery of human CD4^+^ T cells. They also identified AMPK as a potential candidate for enhancing the production of more functional T cells in CAR-T cell therapy [29]. 3. Cell cycle: AMPK can regulate the cell cycle of tumor cells, inhibiting the division and proliferation of tumor cells, and thus affect TIME [36]. Studies have indicated that exogenous introduction of LKB1 into cervical cancer and malignant melanoma with LKB1 deficiency can activate AMPK to upregulate P21 protein expression, leading to G1 phase cell cycle arrest and inhibition of cell proliferation [34]. 4. Inflammatory response: AMPK can regulate inflammatory response and reduce leukocyte infiltration, thus affecting TIME [42]. In rat microglia, transfection of AMPK antisense oligonucleotides or overexpression of dominant inactivated mutant AMPK can potentiate the induction of TNF-α, IL-1β, IL-6, and iNOS by LPS [43]. However, deletion of AMPK in astrocytes and microglia significantly enhances the expression levels of TNF-α, CXCL10, and CCL2 induced by IFN-γ [44]. To sum up, AMPK plays a multifactorial role in TIME. It can regulate the energy metabolism and autophagy mechanism of tumor cells, as well as the activity and inflammatory response of immune cells. It can also achieve metabolic regulation of anti-tumor immunity through molecular crosstalk with major immune checkpoints. However, cellular metabolism functions as a flexible network, and the requirements for metabolism evolve as tumors progress. The initial stage of tumor growth requires nutrient absorption and biosynthesis, the local invasive stage presents additional subtype selective metabolic needs, and the pathway of dependence shifts during the later stage of progression, especially after metastasis and drug resistance. Moreover, AMPK is also a very sensitive energy receptor and regulator. As a result, AMPK exhibits different regulatory mechanisms in different stages of tumor occurrence, development, and even metastasis. However, few existing reports have investigated its mechanisms by stage and situation, resulting in great heterogeneity. Figure 1 illustrates the dual role of AMPK in the TIME. Consequently, we speculated that AMPK plays an important role in regulating energy metabolism at TIME and improving the efficacy of tumor immunotherapy, but it should be tailored to different pathways at different stages of tumor development and manifestation.

## AMPK regulates energy metabolism pathways in cells and TIME

**Fig. 2:**
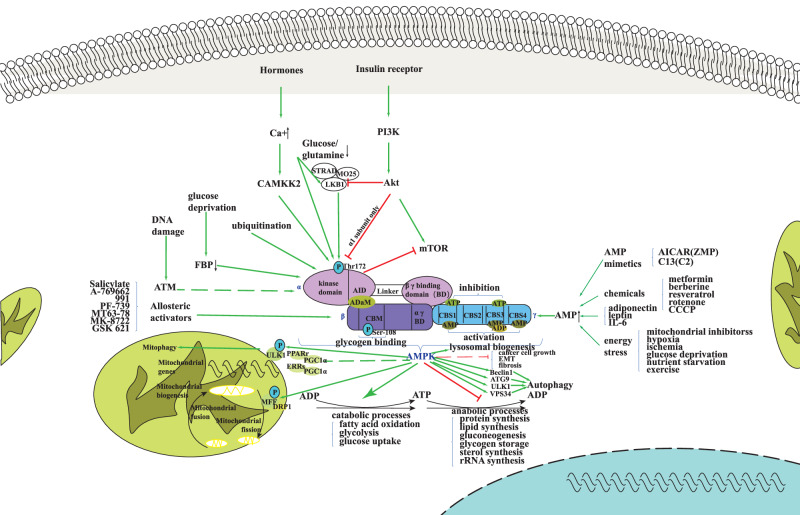
Structure, function, and main regulatory factors of AMPK. AMPK is comprised of three subunits, namely α, β, and γ. It can be activated by CAMKK2, which is regulated by Ca2^+^ concentration, as well as by LKB1, consisting of STRAD, MO25, and LKB1. Additionally, AMPK can be activated by compounds, such as Salicylate and A-769662, or factors that increase AMP levels, including AMP analogues like AICAR, exercise, energy stress such as glucose deficiency, and drugs like aspirin or compounds such as metformin. The primary functions of AMPK are to promote the ATP synthesis process (FAO, glycolysis, glucose uptake, etc.), inhibit the ATP decomposition process (such as protein synthesis, lipid synthesis, gluconeogenesis, etc.), regulate mitochondrial homeostasis, and promote autophagy. AMPK AMP-activated protein kinaseST, PI3K phosphoinositide 3-kinase, CAMKK2 recombinant calcium/calmodulin-dependent protein kinase kinase 2, MO25 mouse protein-25, LKB1 Liver kinase B1, mTOR mammalian target of rapamycin, FBP fructose 1,6-bisphosphatase, ATM Ataxia Telangiectasia Mutated, BD binding domain, CBM carbohydrate-binding module, CBS Cystathionine Beta-Synthase, ATP adenosine 5’-triphosphate, ADP adenosine diphosphate, AMP adenosine monophosphate, CCCP Carbonyl cyanide 3-chlorophenylhydrazone, EMT Epithelial-mesenchymal transition, ATG9 autophagy-related protein 9, ULK1 Unc-51-like kinase, PGC1 peroxisome proliferator activating receptor C-coactivator 1, ERRs Estrogen-related receptors, PPARr peroxisome proliferators-activated receptors, MFF MAC-Forced Forwarding, DRP1 dynamin-related protein 1.

AMPK is a heterotrimer composed of one catalytic subunit (α subunit: by regulating ATP production and consumption, plays a crucial role in the process of energy metabolism of cells) and two regulatory subunits (β subunit: mainly involved in the stability of AMPK, γ subunit: is an important factor in AMPK activation), each of which has multiple isomers (α1, α2, β1, β2, γ1, γ2, γ3) encoded by different genes, resulting in the formation of 12 distinct AMPK complexes with varying subcellular localization functions and substrate specificity [30]. (Table 1: Structure and tissue distribution of AMPK) (Fig. 2: Structure, function, and main regulatory factors of AMPK) The expression of AMPK subunits is widely found and well clear in eukaryotes, but there are great differences among different species, tissues of the same species, and subcellular localization [45].

**Table 1:** Structure and distribution of AMPK.

Enzyme	Subunit	Subtype	Main composition	Main function	Gene	Chromosome	Main distribution
AMPK	α (catalytic subunit)	α1	Catalytic domain the key residue Thr172 for upstream activation	Binding upstream kinase catalyzes AMPK activation	PRKAA1	5p12	Lungs, kidneys, heart, liver and brain
		α2			PRKAA2	1q31	Skeletal muscle, heart, liver, and brain
	β (regulatory subunit)	β1	Glycogene-binding domain; link domain (Linking α - and γ- subunits)	Form AMPK complex	PRKAB1	12q24.1	Liver, few distributed in skeletal muscle
		β2			PRKAB1	1q21.1	Skeletal muscle, a few distributed in the liver
	γ (regulatory subunit)	γ1	Four series CBS domains	Adenine nucleotides can bind to AMPK and change the ATP/AMP ratio accordingly	PRKAG1	12q12-14	Various histiocytes
		γ2			PRKAG2	7q35-36	Various histiocytes
		γ3			PRKAG3	2q35	Skeletal muscle

AMPK is an important regulator of intracellular metabolism and an energy sensor that responds to AMP/ATP ratio changes rather than total phosphorylation levels [30]. When the energy is sufficient, the level of ATP increases and connects to the γ subunit Bateman domain, ATP binds to sites 1,3, and AMP binds to sites 4, inhibiting AMPK activity. Under metabolic stress caused by glucose and amino acid deficiency, elevated AMP binds to the CBS-3 domain of the gamma subunit, directly phosphorylates Thr172 residues of the α subunit(α-172), and binds AMPK to axons on lysosomal membranes, activating AMPK through phosphorylation of α-172 by LKB1 (Liver kinase B1, a cancer suppressor gene encoded by STK11 gene and composed of STRAD, STE20, and MO25) in lysosomes. This activation prevents phosphatase from binding to α-172, inhibiting multiple pathways of dephosphorylation [46], and enhancing AMPK activity [47, 48]. As a result, it reduces the inactivation of AMPK [49]. (Fig. 2: Structure, function, and main regulatory factors of AMPK).

Activated AMPK can also phosphorylate and inhibit ACC1 and ACC2 (fatty acid synthesis rate-limiting enzymes that catalyze the first step of lipid synthesis), thereby inhibiting the synthesis of fatty acids (FA) [50]. It can also increase the translocation of FA transporter CD36 to the plasma membrane, allowing existing FA to enter the cell, increasing the utilization of mitochondria, and increasing FAO [51, 52]. AMPK phosphorylates TXNIP and TBC1D1, increases plasma membrane localization of GLUT1 and GLUT2, increases glucose transportation, stimulates glucose utilization, increases glucose uptake by cells [53], and boosts glycolysis by phosphorylating PFKFB3 (affecting the activity of the glycolytic rate-limiting enzyme PFK1) [54]. Tumor cells can promote their own growth and development by inhibiting immune cell glycolysis to escape immune killing. Yachi, P. P. team [55] found that tumor metabolites can inhibit the glycolysis of CD8^+^T cells, resulting in inhibition of cell proliferation, cytokine production, and cytotoxicity, resulting in damage to the anti-tumor effect of CD8^+^T cells. Activating AMPK of T cells during TIME can enhance their activity and function. AMPK can also activate glycogen phosphorylase by the process of phosphorylation, activate glycogen decomposition [56, 57], inhibit the phosphorylation of GYS1 and GYS2, inhibit glycogen synthesis, and prevent glycogen storage [58].

When it comes to energy metabolism, of course, mitochondria, the main site of oxidative decomposition, cannot be reduced. Activation of AMPK can promote mitochondrial biosynthesis. AMPK can phosphorylate PGC1α (peroxisome proliferator activating receptor C-coactivator 1α) and make it have transcriptional activity through positive feedback regulation. AMPK can also increase the concentration of NAD to increase SIRT1 (acetylase) mediated PGC1α deacetylation and activate PGC1α, thus regulating mitochondrial biosynthesis [59]. Mitochondria are the primary sites of ROS (reactive oxygen species) production in cells and are vulnerable to oxidative damage. Autophagy is a process through which cells digest and recycle themselves [59]. When mitochondria become damaged, aged, or dysfunctional, autophagy plays a crucial role in clearing and repairing these mitochondria [59]. This process is essential for maintaining the protein and energy balance of cells, regulating cell physiological function, and preserving cell homeostasis. Therefore, autophagy plays a crucial role in maintaining the body’s energy metabolism [59]. AMPK also can promote waste disposal and decomposition by regulating autophagy, releasing decomposition products that can be used to produce energy. Activated AMPK reduces T cell immune fatigue by reducing ROS and minimizing the accumulation of metabolic waste. On the contrary, autophagy changes the pH of TIME by removing acidic metabolites, affecting the growth and survival of TIME cells.

In summary, AMPK can influence the acidity of TIME and the metabolic activity of various immune cells by regulating energy metabolism and autophagy, thus improving the efficacy of tumor immunotherapy.

In addition to energy stress, oxidative stress can also directly phosphorylate Cys residues in AMPK, activate AMPK, up-regulate antioxidant genes, and phosphorylate activate Forkhead Box O (Fox O) to reduce superoxide levels [60]. In HEK293 and lung cells, H2O2 has been shown to induce oxidation of α-CYS-2999/304 and S-glutathione acylation, thereby activating AMPK [61], the AMPK-ACC1/2 axis inhibits FAS and activates FAO, detoxifying ROS by maintaining NADPH [62] and GSH levels. Apart from indirectly activating AMPK via increasing AMP [33], some studies have shown that ROS can also regulate AMPK activity through direct post-translational modification [63]. AMPK can promote oxidative phosphorylation of macrophage mitochondria by activating NADPH oxidase in macrophages, increasing intracellular ROS levels, inhibiting NADH dehydrogenase activity, enhancing mitochondrial membrane potential, and promoting electron flow in the electron transport chain [64].

AMPK can also regulate the activity and expression of COX (cytochrome c oxidase) through direct phosphorylation of subunits of the COX or indirect regulation of COX-related transcription factors. As a result, it can regulate the metabolic balance of TIME and maintain the activity and function of immune cells [65]. Studies have shown that AMPK activator AICAR (5-aminoimidazole-4-carboxamide-1-β-D-ribofuranoside) significantly increases the COX activity in immune cells, but this effect is antagonized by AMPK inhibitors [66]. A certain study revealed that AMPKα1 increases mitochondrial ATP production by phosphorylating COX I, providing more energy to immune cells in TIME [67].

AMPK can not only regulate the metabolic state of tumor cells but also increase the diversity and flexibility of the internal metabolism of tumor cells, so as to increase the drug resistance of tumor cells and improve the difficulty of treatment [68]. It can also regulate the metabolic state of immune cells, maintaining their activity and functioning to reduce tumor metastasis and enhance immunotherapy for tumors. Therefore, AMPK is a “double-edged sword”. We are highly looking forward to some further studies to find out which pathway and when to activate AMPK in TIME. AMPK can better inhibit the growth and metabolism of tumor cells, thereby reducing the abnormal metabolism and immunosuppression level at TIME, and at the same time, enhance the activity and infiltration of CD8^+^T cells in TIME, leading to enhance the immunotherapeutic effect of the tumor, enhancing the negative regulatory effect of macrophages and dendritic cells (DC cells), inhibiting the immunosuppressive effect of TIME, and ultimately promoting the immunotherapeutic efficacy of a tumor.

**Fig. 3:**
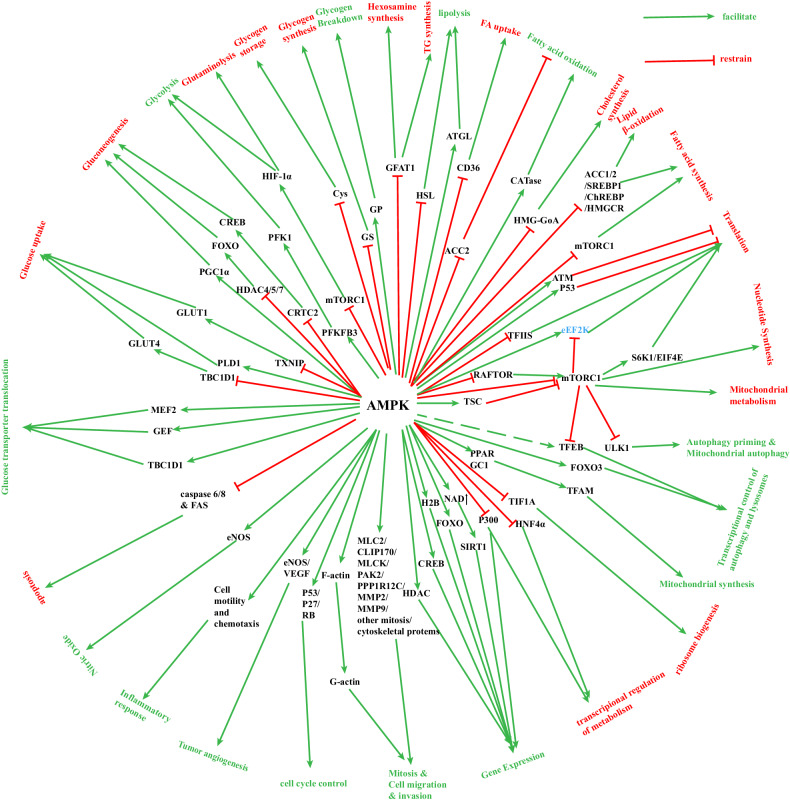
The regulation of AMPK on energy metabolism, mitochondrial homeostasis, and autophagy. AMPK activation regulates a variety of energy metabolic pathways. Green arrows represent up-regulated processes while red blockers represent down-regulated processes. Text in green font indicates biological processes that are mainly upregulated by AMPK, while text in red font indicates biological processes that are mainly downregulated by AMPK. These processes can be classified into glucose metabolism, lipid metabolism, protein metabolism, mitochondrial homeostasis, autophagy, and ribosome regulation. It is evident that AMPK plays a central role in energy metabolism and oxidative metabolism. AMPK AMP-activated protein kinase, GFAT1 Glutamine fructose-6-phosphate amidotransferase 1, HSL hormone-sensitive triglyceride lipase, ATGL adipose triglyceride lipase, CD36 Platelet glycoprotein 4, ACC2 acetyl-CoA carboxylase 2, mTORC1: mechanistic target of rapamycin complex1, ATM Ataxia Telangiectasia Mutated, P53 Cellular tumor antigen p53, eEF2K Eukaryotic extension factor 2 kinase, TSC TSC Complex Subunit 1, S6K1 Ribosomal S6 kinase 1, EIF4E eukaryotic initiation factor 4E, ULK1 Unc-51-like kinase, PPAR peroxisome proliferators-activated, GC1 Glume Coverage 1, TFAM Transcription Factor A, Mitochondrial, TIF1A Transcription Intermediary Factor 1-Alpha, HNF4α hepatocyte nuclear factor 4alpha, SIRT1 Silent Mating Type Information Regulation 2 Homolog 1, NAD nicotinamide adenine dinucleotide, H2B: histone 2 B, CREB: cyclic-AMP response binding protein, HDAC histone deacetylase, MLC2 Myosin Light Chain 2, VEGF vascular endothelial growth factor, eNOS endothelial nitric oxide synthase, FAS fatty acid synthesis, TBC1D1 Tre-2/BUB2/cdc 1 domain family 1, GEF GMP exchange factor, MEF2 myocyte enhancer factor-2, PLD1 Phospholipase D1, GLUT Glucose Transporter, TXNIP thioredoxin-interactingprotein, PFKFB3 6-Phosphofructo-2-Kinase/Fructose-2,6-Biphosphatase 3, PFK1 Phosphofructokinase-1, HIF-1α Transcription Intermediary Factor 1-Alpha, GS Glutamine synthetase, GP Glycogen phosphorylase.

In recent years, researchers have discovered that activated AMPK can regulate cell metabolism and cytokines, and significantly affect TIME by blocking immune checkpoint molecules PD-1 and CTLA-4 [69]. Immune checkpoint molecules are costimulatory cell surface receptors expressed on the surface of several immune cells, which bind to corresponding ligand molecules and in turn prevent an immune attack on autoantigens [70]. However, tumor cells exploit this feature of immune checkpoints to evade immune attack, giving them a survival advantage. Immunotherapeautic drugs are designed to suppress these immune checkpoints, rendering tumor cells vulnerable to immune attack [71]. PD-1 receptor is currently used as a frequent treatment for lung cancer [4]. AMPK activation leads to phosphorylation of PD-L1 at Ser283 and disrupts its interaction with CKLF-like CMTM4, resulting in the degradation of PD-L1 [72]. AMPK also enhances ER (endoplasmic reticulum)-related degradation of PD-L1 [3, 72]. Shiravand Y et al. [73] have demonstrated that activating AMPK can enhance the activation and proliferation of T cells and improve tumor immune response by inhibiting PD-L1. On the other hand, Dai et al. [72] reported that AMPK agonists enhanced the efficacy of anti-CTLA-4 immunotherapy and improved overall survival in genetically identical mouse tumor models. In addition, it has also been reported that the combination of AMPK activator and anti-VEGF /PD-1 drugs can be used as dual-targeted therapy for ovarian cancer [69]. A recent report by Pokhrel et al. [74] suggested that AMPK blocks PD-1 expression through the HMGCR-p38 MAPK-GSK3B signaling pathway, thereby promoting anti-tumor immunity. Tryptophan starvation inhibits CD8^+^T cell effector function and stimulates CD4^+^ regulatory T (Treg) cell function, resulting in a powerful immunosuppressive effect that affects CTLA4 and PD1/PD-L1 pathway-mediated immune checkpoints, resulting in tumorigenic TIME [75]. Nevertheless, an activation of AMPK can change the above environment, converting tumorigenic TIME become tumor suppressive TIME [74]. It has also been reported that AMPK activator has synergistic anti-tumor effects with anti-PD-1 antibodies, anti-CTLA-4 antibodies, or HMGCR inhibitors in mouse tumor models [3]. Recent studies have also shown that AMPK activation can enhance another key immune checkpoint molecule LAG-3 (lymphocyte activation gene 3) inhibitor, further regulate T cell metabolism and function, and enhance immune efficacy against tumors [76]. Collectively, these findings highlight the central role of AMPK in inhibiting major immune checkpoints. Figure 3 shows the AMPK-mediated modulation of each component of TIME and the associated signaling pathways.

## AMPK enhances immunotherapeutic efficacy by regulating metabolic reprogramming in TIME

The metabolic state of immune cells is crucial to their functional plasticity [40, 76]. The malignant progression of tumors and chemotherapy resistance are the manifestations of metabolic reprogramming of immune cells that occur in TIME [77]. Due to the multiple functions of AMPK in regulating basic cell activities, it contributes to tumor metabolic transformation and controls the metabolic plasticity of various immune cell types in TIME, thereby enhancing anti-tumor immune response [78].

T cell activation in TIME is controlled by various metabolic pathways [32]. The metabolic adaptability of T cells is crucial for anti-tumor immunity, and AMPK plays a significant role in regulating the metabolism and activity of T cells in the immune system. AMPK activation can regulate the energy metabolism of T cells, such as limiting anabolism and promoting mitochondrial homeostasis to allow continuous glycolysis, enhancing their activation state and killing capacity [79, 80]. Glucose starvation also promotes AMPK activation in T cells independently of AMP/ADP by reducing intracellular concentrations of fructose-1, 6-diphosphate [81]. Meanwhile, inhibition of AMPK prevents activated T cells from increasing glucose and amino acid uptake [82]. Furthermore, AMPK also promotes FAO through activation, enhances glutamine dissolution and mitochondrial OXPHOS, regulates metabolic reprogramming of T cells, and enhances T cell activity [83]. Cytotoxic CD8^+^ T cells play a crucial role in tumor immune monitoring and induce anti-tumor immune response by secreting IFN-g and granzyme B (GZB) [84]. It has been proposed that AMPK-mediated changes in T cell metabolism not only improve the metabolic adaptability of CD8^+^ T cells to metabolic stress but also is central to determining whether CD8^+^ T cells produce long-lived memory cells (memory CD8^+^ T cells, Tmem, TM) or ultimately differentiate effect CD8^+^ T cells (Teff, TE) [85]. This idea was derived from experiments that demonstrated that in vivo treatment of mice with the mTOR inhibitor rapamycin increased TM production by accelerating the conversion of TE to the memory pool [86], and experiments with defective AMPK mice had the same conclusion and results [87]. Although AMPK-deficient CD4^+^ T cells can develop into Th1 and Th17 lineages and retain their effector function, the total number of anti-gene specific CD4^+^ T and CD8^+^ T cells in the body is reduced, resulting in a weakened immune response [88]. Studies have also shown that treating mice with metformin activates AMPK and enhances TM production [85]. The findings from the above two observations may be related. As reported, metformin interferes with oxidative phosphorylation-mediated ATP synthesis by inhibiting respiratory chain complex I, thereby leading to an increase in the cellular AMP/ATP ratio. This energy stress triggers LKB1-dependent activation of AMPK, which then phosphorylates TSC2 and Raptor to coordinate the inhibition of mTORC1 activity [89]. AMPK can also restrict the expansion and activity of MDSCs (Myeloid suppressor cells, an immunosuppressive type of immune cell, are pathologically activated in various tumor types. MDSCs promote tumor progression and enhance tumor cell survival, angiogenesis, invasion, metastasis, and the production of immunosuppressive cytokines such as IL-10 and TGF-b.) by inhibiting JAK-STAT, NF-kB, C/EBPb, CHOP, and HIF-1a pathways, and weaken MDSCs from preventing CD8^+^ T cells from entering the tumor site [90–93]. The AMPK-mTOR signaling pathway is essential for T cell development in the thymus, peripheral homeostasis, and differentiation of effector CD4^+^ Th1, Th2, and Th17 cells as well as cytotoxic CD8^+^ T cells [94]. The AMPK-mTOR axis is also responsible for TM production and TE adaptation to nutritional stress [95, 96]. Moreover, LKB1-AMPK signaling pathway plays a central role in regulating cell metabolism, proliferation, and survival in response to changes in nutrient and energy requirements, and promotes metabolic reprogramming of T cells by stimulating catabolic pathways and ATP production in TIME, thus contributing to the differentiation and function of T cells under energy stress [97]. Thus, in TIME, AMPK’s metabolic flexibility helps T cells adapt to nutrient-poor environments.

In addition to T cells, B cells, the main force of humoral immunity, are also an important part of the immune system. AMPK also affects the metabolism and activity of B cells, which in turn affects the function of B cells in immune response. Activated AMPK can regulate metabolic pathways within B cells [35], such as improving nutrition and energy utilization by B cells through increased glycolytic pathways, glucose transport and metabolism, and mitochondrial respiration. Studies have demonstrated that AMPK activation can increase the absorption and utilization of FAO and glucose by B cells, enhance the proliferation and migration ability of B cells in vivo, and thus inhibit tumor growth, which has been widely a concern in tumor immunotherapy [98]. A recent study has shown that AMPKα1 induces B cells to perform autophagy, thereby eliminating unwanted organelles and proteins, maintaining cell homeostasis and function, and promoting B-cell mitochondrial homeostasis. It can also increase protein synthesis and antibody class conversion, which not only facilitates the differentiation and proliferation of B cells but also increases the persistence of B cell memory [99]. AMPK influences the immune response in TIME by regulating cytokine secretion and antibody production in B cells [100]. However, there is limited literature on the role of AMPK in regulating B cell metabolism and function and humoral immunity. Therefore, assessing the mechanisms of AMPK-mediated humoral immune regulation of various tumor types will help expand existing knowledge.

NK cells are the frontline defense of natural immunity and play an essential role in anti-tumor immunity. However, due to the complex immune regulation and metabolic processes in TIME, most advanced tumors normally escape the killing of NK cells. Consequently, can restoration of the function of NK cells effectively prevent tumor escape? A recent report [101] showed that the formation of immune synapses is a key step in the dissolution and killing of tumor cells by NK cells; however, NK cells lose their surface membrane prominences and cannot recognize tumor cells in TIME, making them lose their anti-tumor function. They also developed a mass spectrometry technique for single immune cell membranes, and this mouse study revealed that the loss of sphingomyelin (SM), a major component of NK cell membranes, is the primary cause of NK cell surface projection loss. Further using the co-culture system, they revealed that the main reason for the decrease in SM level of NK cells in tumors was the dysregulation of serine metabolism. Additionally, regulating serine metabolism and further blocking SM catabolism can not only promote the formation of protruding processes but also have synergistic anti-tumor effects with immune checkpoint inhibitors. It is worth mentioning that AMPK has been shown to regulate the serine metabolic pathway in cells through various ways [102]. For example, AMPK can decrease its catalytic activity by phosphorylating serine carboxymethyl transferase (MAT), subsequently reducing serine synthesis. It also influences serine metabolism by modulating the mTOR signaling pathway. Inside the cell, mTOR is an important regulator of protein synthesis, which can regulate the utilization of amino acids like leucine and serine [103]. AMPK inhibits the activity of the mTOR signaling pathway, thereby reducing the serine consumption of cells [102]. Recent studies have also found that AMPK regulates serine/threonine phosphatase, a key enzyme in cell metabolism, thereby affecting cell utilization of serine and threonine [103]. The aforementioned studies uncover a novel mechanism of tumor immune escape and provide a fresh perspective and a new target for tumor immunotherapy based on NK cells. Furthermore, the activation of AMPK can directly promote the production of NK cells, increase their capability to traverse the blood-brain barrier and blood vessels of tumor tissues, and augment their cytotoxic effect to bolster the immune killing ability of tumor cells [104]. In conclusion, AMPK regulates the activity of NK cells and apoptosis of tumor cells in TIME through a variety of mechanisms, thus affecting the effect of tumor immunotherapy. Therefore, AMPK may become a promising target for tumor immunotherapy, which is expected to improve the efficacy of immunotherapy.

In tumor immunotherapy, the activation of AMPK has been found to affect the phenotypic transformation and function of macrophages by focusing on solving metabolic problems such as FAO and mitochondrial metabolism, thereby enhancing the attack force of the immune system and reducing immune escape, ultimately enhancing the tumor-killing ability [105]. Macrophages are one of the most abundant immune cell types in TIME and play a crucial role in the regulation of TIME due to their functional plasticity leading to their anti-tumor and pro-tumor functions in different environments. Macrophages play an important role in anti-tumor immunity through phagocytosis and antigen presentation, mainly being divided into tumor-killing M1 type and tumor-promoting M2 type [106]. However, activation of AMPK can increase the glycolysis rate through the Akt-HIF1α-mTOR pathway, thereby polarizing macrophages towards the M1 subtype. Additionally, M1 macrophages secrete a series of anti-tumor factors, such as cytokines IL-12 and IL-18, thereby inhibiting tumor growth [107]. At the same time, they also have the ability to recognize and destroy tumor cells, thereby enhancing the effectiveness of tumor immunotherapy. Chiang et al. [108] showed that metformin inhibited M2-type polarization of macrophages in breast cancer cells by activating AMPK, which was also supported by Say D et al. [109]. Contrarily, M2 macrophages, another common macrophage type, secrete a series of immunosuppressive factors, such as IL-10 and TGF-β, which inhibit the activity of CD8^+^ T cells and promote tumor growth [110]. Furthermore, AMPK inhibits HIF-1a and NF-kB by deacetylating proteins such as SIRT1 and NAD+, influencing M1/M2 polar mitochondrial biogenesis in macrophages [111]. Activation of AMPK can inhibit the accumulation of metabolites such as pyruvate and lactic acid produced by macrophage metabolism, thereby reducing the acidification effect of TIME and making immune cells more active [112]. Moreover, activation of AMPK also enhances the immune response by promoting the autophagy of macrophages and eliminating metabolic wastes and antigens [113]. Previous reports have also demonstrated that AMPK is involved in molecular crosstalk between macrophages and cytokines [114].

In summary, AMPK has the ability to reduce the immunosuppression of TIME and improve the effectiveness of tumor immunotherapy by regulating the metabolism and activity of T cells, B cells, NK cells, macrophages, and other immune cells of TIME. However, TIME is made up of multiple immune cells, all of which play a vital role in disease progression, and targeting just one type of cell is insufficient to alter the entire TIME and thus eradicate the tumor. The specific mechanism by which AMPK enhances the efficacy of tumor immunotherapy by regulating metabolic reprogramming still needs to be further studied and elucidated in order to obtain significant breakthroughs and improvements in tumor therapy.

## Crosstalk between AMPK and tumor immunity in the microenvironment: implications for anti-tumor activity

Numerous studies have demonstrated that metformin can significantly reduce the risk of cancer in diabetic patients because metformin can induce energy stress by inhibiting the mitochondrial respiratory chain complex I [115, 116], which leads to the increase of AMP/ATP ratio and activation of AMPK [31, 32]. Mice model experiments have shown that activation of AMPK regulates the polarization of macrophages and enhances their phagocytosis and lysosomal functions [117]. At the same time, the autophagy process of tumor cells is increased, which delays the growth and attack of tumor cells [117]. Moreover, metformin can induce AMPK-dependent metabolic regulation of T cells in TIME, thereby enhancing their activity and increasing the quantity and force of TM, thereby enhancing genetic immunity and tolerance, and thereby increasing the response and efficiency of tumor immunotherapy [118]. Simultaneously, it can inhibit the metabolic activity of Treg cells, regulate the expression of surface markers and a variety of cytokines, and further inhibit the proliferation and function of Treg cells, thereby reducing tumor immune escape [119] and improving the sensitivity and therapeutic effect of tumor cells. Nonetheless, when the LKB of the mouse liver was knocked out of, liver AMPK was no longer activated by metformin, and metformin also lost its anti-hyperglycemic effect [120]. The inhibitory effect of metformin on glucose production was still observed in isolated hepatocytes of mice with AMPK catalytic subunit knocked out [120]. This experiment is consistent with results from another genetic model showing that AMPK-deficient T cells are more sensitive to energy stress in vitro than LKB1-deficient T cells, but can still develop normally in vivo thymus [121]. Therefore, LKB1 is an important regulator of AMPK in energy-stressed T cells [122], so the therapeutic effect of metformin-mediated AMPK may not be ideal in LKB1-deficient tumor patients.

A-769662 is a thienopyridine compound that activates AMPK through AMP allosteric activation rather than phosphorylation of α-172 [123]. Therefore, A-769662 can activate AMPK in mutant cells [124]. Is A-769662 expected to provide A boon for patients with LKB1-deficient tumors? The action of A-769662 can involve multiple members of TIME, including tumor cells and immune cells. A-769662 activates AMPK by directly binding to different domains of the AMPKβ subunit [125], inhibiting tumor growth and proliferation, and promoting apoptosis and autophagy. At the same time, mouse models have shown that A-769662 can also stimulate the metabolism and phagocytosis of macrophages and accelerate the clearance of tumor cells [126]. In addition to macrophages, A-769662 regulates the action of DC cells through the AMPK signaling pathway, increasing their antigen presentation and T cell activation. Collectively, A-769662 also activates AMPK and inhibits the production of immunosuppressive factors in TIME, thereby enhancing the immunotherapy effect of TIME.

Salicylate was found to activate AMPK by directly binding to the synthetic activator A-769662 at the same site, causing conformational changes that both inhibited α172 dephosphorylation and induced allosteric activation [127]. More commonly used clinically than A-769662 is acetylsalicylic acid (trade name aspirin), which is easier to take orally. It has been shown that plasma AMPK concentration increases rapidly after oral high-dose aspirin [124]. When aspirin was administered to normal mice, FAO was enhanced, but knocking out AMPKβ1 eliminated this response in mice [124]. Additionally, aspirin is a commonly used drug in clinical practice, and studies have shown that it does play a positive role in tumor prevention, which is attributed to AMPK mediation in further studies [128]. A prospective study of 135,965 subjects by Cao et al. found that compared with non-regular aspirin users, regular aspirin users had a 15% (relative risk (RR) = 0.85) lower risk of digestive tract tumors and a 19%(RR = 0.81) lower risk of colorectal cancer [129]. Analysis of 52 cohort studies by Elwood et al. showed that regular aspirin use was associated with a 24% (HR = 0.76) lower risk of colon cancer death, a 13% (HR = 0.87) lower risk of breast cancer death, and an 11% (HR = 0.89) lower risk of prostate cancer death [130]. In conclusion, regular aspirin use can both reduce the risk of developing tumors and reduce the risk of death in cancer patients. Some researchers speculate that the possible reason is that salicylate activates AMPK by reducing intracellular ATP levels [131], promotes Treg cell death through PI3K-Akt-mTOR pathway, and inhibits its proliferation and function, thereby reducing tumor immune escape [132]. In addition, aspirin can also inhibit the expression of immune checkpoint in TIME [133], thereby enhancing the body’s killing effect on tumor cells and the phagocytosis of macrophages [134].

In addition to the common clinical aspirin, it is claimed that the anti-inflammatory mechanism of the anti-cancer drug methotrexate is also accomplished by activating AMPK. ZMP, which is an intermediate of purine nucleotide biosynthesis synthesized by AICAR in cells, is metabolized into IMP (inosine phosphate) in two steps. Methotrexate is a folate analogue that inhibits enzymes that use tetrahydrofolate. Therefore, it was found that when breast cancer cells were incubated with AICAR, methotrexate caused an increase in ZMP accumulation and enhanced AMPK activation [135]. In human leukemia cell lines, pemetrexel (a highly selective AICAR invertase inhibitor), another anti-folate drug commonly used in lung cancer, causes ZMP accumulation and AMPK activation even in the absence of AICAR [136]. This suggests and verifies that some of the anti-cancer effects of antifolate drugs may be mediated by AMPK. Methotrexate increases the intracellular AMP/ATP ratio, thereby activating AMPK [137]. Once AMPK is activated, it leads to an energy imbalance within the cell, which inhibits tumor growth and proliferation [138]. The effects of methotrexate are not limited to tumor cells but also involve other cells and signaling pathways of TIME. For instance, methotrexate inhibits the proliferation and angiogenesis of vascular endothelial cells at TIME [137], thus affecting the nutrient supply of tumor cells. In addition, methotrexate can also suppress the production of immunosuppressive factors in TIME [139], promote the activation and infiltration of tumor immune cells, and increase the effect of tumor immunotherapy. When activated by methotrexate, AMPK can inhibit mast cell activation and release, reduce inflammatory response and immunosuppression in TIME, and promote mast cell apoptosis, thus inhibiting tumor growth and metastasis [139]. Moreover, methotrexate can also promote T cell proliferation and function in TIME, improve immune activity, and thus enhance tumor immunosuppression [140]. In conclusion, the mechanism of action of methotrexate involves indirect regulation of AMPK and has regulatory effects on multiple components of TIME, thereby affecting tumor growth and metastasis and improving tumor immunotherapy efficacy.

In brief, several AMPK activators (i.e., metformin, A769662, salicylate, methotrexate, etc.) have been displayed to be anti-tumor, and almost all of them are related to AMPK mediating. Nonetheless, long-term, large-scale clinical trials of these drugs are necessary to verify their efficacy, safety, and precise mechanisms of action. Tests utilizing in vivo genetic models of AMPK deficient immune cells are also needed to provide more valuable reference data for potential clinical applications.

## Outlook

The metabolic reprogramming that regulates TIME is a promising therapeutic strategy regardless of cancer type. Enhancing AMPK activity can affect TIME and enhance tumor immunotherapy by regulating metabolic reprogramming. However, due to the large number of AMPK members and their mutual influence, the regulation of AMPK is limited to a certain extent. We speculate whether the major upstream LKB1 of AMPK or its major downstream mTOR can be used as biomarkers to predict the efficacy of immunotherapy and expand the benefit population of immunotherapy with its corresponding activator or inhibitor. AMPK, as an essential protein kinase, is still being studied extensively, and its role in energy metabolism, cell proliferation, immune regulation and other aspects is becoming more and more obvious, and it is also involved in various fields such as human lifespan and antioxidants. Collectively, we have a strong reason to believe that with the deepening and more investigation in the research on AMPK and its upstream and downstream major factors, its application in tumor immunotherapy and other fields will show tremendous potential, to make vast contributions to human health and life.
